# Epidemiologic Study of Syrian Refugees Underwent Surgery Due to Fracture in a Tertiary Reference Hospital in Turkey

**DOI:** 10.7759/cureus.13323

**Published:** 2021-02-13

**Authors:** Adem Sahin, Anıl Agar, Cafer Ozgur Hancerli, Bulent Kilic, Deniz Gulabi, Cemil Erturk

**Affiliations:** 1 Orthopaedics and Traumatology, Kanuni Sultan Süleyman Training and Research Hospital, University of Health Sciences, Istanbul, TUR; 2 Orthopaedics and Traumatology, Saglik Bilimleri University, Kanuni Sultan Suleyman Training and Research Hospital, Istanbul, TUR; 3 Orthopaedics, Kanuni Sultan Süleyman Training and Research Hospital, Istanbul, TUR

**Keywords:** epidemiology, fracture, operation, syrian refugees

## Abstract

Aim

This study aims to analyze the spectrum, management, and outcome of Syrian refugees’ fracture over four-year period, highlighting challenges in management and follow-up.

Methods

This was a retrospective review of Syrian refugee patients operated for fractures at our centre from January 2015 to January 2019. The patients were evaluated for age, gender, mechanism of injury, location and type of fracture, presence of accompanying injuries, surgical technique, complications, mortality and morbidity. The comparison of complications and postop outpatient clinic controls between Turkish citizens and Syrian refugees were also evaluated.

Results

The study included a total of 455 patients comprising 281 adults (202 males, 79 females) with a mean (SD) age of 41.1 (19.3) years and 174 children with a mean age of 8.8 (4.9) years. The trauma mechanism was most commonly fall in both adult and pediatric patients (86.6% / 73.5%). Whilst lower limb fractures were more common in adults (73.7%), upper limb fractures were more common in children (63.4%). The presence of accompanying trauma was determined in 21 (7.5%) adults and 10 (5.7%) children. Multiple fractures were determined in 12 (4.3%) adults and eight (4.6%) children. Plate fixation (PF) was most used in 137 (48.8%) adult patients and K-wire augmentation was used in 75 (43.1%) pediatric patients. Out of the 455 patients, 41 (14.6%) adults and 13 (7.3%) children developed complications. Whilst three adult patients were died during follow-up, no deaths were recorded in the pediatric patients. Complication rate was 54/455 in Syrian refugees and 32/455 in citizens. It was observed that the complication was significantly higher in immigrants (p: 0.017). Sixty-five (14.2%) Syrian immigrants did not come to the outpatient clinic control at all or once, while this rate was 29/455 (6.3%) for Turkish citizens (p = 0.012).

Conclusion

Inadequate living conditions and lack of communication faced by refugees reduce the rate of patient follow-up and negatively affect the results of orthopedic trauma.

## Introduction

Since the beginning of the Syrian conflict in March 2011, more than 10 million Syrians have had to leave their homes and country and have sought refuge in neighboring countries [1]. Turkey is hosting 3.5 million people, which is the largest number of refugees in the world [2].

Refugees who lived in the camps in the first years later started to move to cities later, and of those living outside the camps, the majority of refugees settled in Istanbul at the rate of 24.8% [3]. Initially there was a low rate of utilization of healthcare services by the Syrian refugees living outside the camps, which could be attributed to insufficient information about how to use the healthcare services in Turkey, communication barriers or lack of a state-issued identity number [4]. Over the years, such problems have been resolved with the adaptation process, language learning and the help of the state.

Turkey has provided Syrian refugees the opportunity to benefit from healthcare services provided to Turkish citizens at every level. Healthcare services are provided free of charge to Syrian refugees inside and outside the refugee camps.

Although our hospital is a tertiary center, it is located in a region with a dense population of Syrian refugees. The hospital serves a population of 3.5-4 million, of which approximately 10% is Syrian refugees.

The aim of the present study is to epidemiologically evaluate Syrian refugees who were operated due to fractures.

## Materials and methods

A retrospective evaluation was made of 640 Syrian refugee patients who presented at the emergency department and were operated on because of orthopedic trauma between January 2015 and January 2019. The inclusion criteria were that the patients were Syrian refugees, surgery was because of any orthopedic fracture, had regular medical records, and agreed to participate in the study. Patients with pathological fractures, non-traumatic orthopedic diseases (developmental hip dysplasia, osteoarthritis, etc.), and non-fractured orthopedic trauma (tendon injury, septic arthritis, etc.) and whose records could not be reached were excluded from the study. As a result, 455 Syrian patients were included in the study.

The patients were divided into two groups as adults and children (<18 years). Adult patients were then sub-divided as female and male, and these three groups were evaluated. The diagnoses were divided into three main groups according to the fractured part of the body as the upper limb, lower limb, and pelvis/spine sections.

The age, gender, and trauma mechanisms of the patients were recorded. Patients consulted by other medical branches due to additional injury were enrolled. The open fractures were recorded according to the Gustilo Anderson classification [5]. Patients who were urgently operated on within the first 12 hours were identified.

ASA scores (American Society of Anesthesiologists) of patients were recorded. The length of hospital stay (days) and the orthopedic materials (plate, k-wire, nails, etc.) used in the surgical orthopedic interventions were also recorded.

In order to evaluate the treatment compliance of the patients after discharge, the number of follow-up visits to the outpatient clinic was examined. Complications that developed in the patients and further operations performed were recorded.

Turkish citizens and Syrian immigrants groups were separated and analyzed by 1:1 propensity score matching (PSM), and sample logistic regression analysis was performed using SPSS and R programs. Selected sociodemographic variables used for PSM included age, sex, and postop outpatient clinical controls.

## Results

Patient characteristics

All demographic and clinical characteristics of patients are listed in Table 1. A total of 455 patients were included in the study, comprising 281 adults (M: 202 / F: 79) and 174 children (M: 128 / F: 46). The mean (SD) age of the patients was 41.1 (19.3) years in adults and 8.8 (4.9) years in children. In the gender sub-groups of the adult patients, the mean age of female patients was 52.9 (19.4) years which was significantly higher than the mean age of the male patients (36.5 [17.2] years).

**Table 1:** Demographic distribution of patients according to age group

	Adult (n = 281)	Child (n = 174)	p
Age (mean SD)	41.1 (19.3)	8.8 (4.9)	
Gender (M/F)	202/79	128/46	
Hospitalization period (mean SD)	5.4 (5.1)	3.1 (3.4)	0.001
Hospitalization period in ICU (mean SD)	0.24 (0.67)	0.02 (0.22)	
Injury type	n (%)	n (%)	
Fall / Fall from heights	196 (69.7)	131 (75.2)	
Traffic accident	65 (23.1)	39 (22.4)	
Gunshot	9 (3.2)	1 (0.5)	
Fight	2 (0.7)	0 (0)	
Work accident	9 (3.2)	3 (1.7)	
Injury location			
Upper extremity	56 (19.9)	97 (55.7)	
Lower extremity	213 (75.8)	76 (43.7)	
Axial skeleton	12 (4.3)	1 (0.6)	
Fracture location	n (%)	n (%)	
Humerus/Clavikula	18 (6.4)	71 (40.8)	
Forearm	19 (6.8)	22 (12.6)	
Femur	86 (30.6)	50 (28.7)	
Tibia	64 (22.4)	22 (12.6)	
Hand/Wrist	20 (7.1)	3 (1.7)	
Foot/Ankle	54 (19.2)	5 (2.9)	
Patella	7 (2.5)	0 (0)	
Pelvis	11 (3.9)	1 (0.6)	
Spine	2 (0.7)	0 (0)	
Accompanying trauma	21 (7.5%)	10 (5.7%)	0.568
Multiple fracture	12 (4.3%)	8 (4.6%)	0.981
Treatment	n (%)	n (%)	
Intramedullary Nail	42 (14.9)	7 (4.0)	0.001
Plate	137 (48.8)	44 (25.3)	
Screw	16 (5.7)	10 (5.7)	
Hemi/Total Hip Prosthesis	23 (8.2)	0 (0)	
Proximal Femoral Nail/Dynamic Hip Screw	27 (9.6)	0 (0)	
K Wire/Cerclage	13 (4.6)	75 (43.1)	
External Fixator	17 (6.0)	2 (1.1)	
Titanium Elastic Nail	0 (0)	36 (20.7)	
Amputation/Replantation	4 (1.4)	0 (0)	
Posterior Instrumentation	2 (0.7)	0 (0)	
Complication	41 (14.6%)	13 (7.5%)	0.02
Gustilo classification	n (%)	n (%)	
Type 0	264 (94)	171 (98.3)	
Type 1	7 (2.5)	2 (1.1)	
Type 2	4 (1.4)	0 (0)	
Type 3	6 (2.1)	1 (0.6)	
Reoperation	39 (13.9%)	32 (18.4%)	0.23
ASA scores	n (%)	n (%)	
ASA 1	174 (61.9)	166 (95.4)	
ASA 2	66 (23.5)	7 (4)	
ASA 3	41 (14.6)	1 (0.6)	

Trauma type, location, and mechanism

The most common mechanism of trauma was falling from heights in both adults and children (62.3% / 73.6%). Lower limb fractures (73.7%) were more common in adults and upper limb fractures (63.4%) were more common in children. Whilst, fractures were most commonly reported in the femur region followed by tibia and foot/ankle in adults, respectively, supracondylar humerus fractures were most common in children (n: 46, 26.4%). The presence of accompanying trauma such as abdomen-thorax-head and genitourinary system injuries were determined in 21 (7.5%) adults and 10 (5.7%) children. Multiple fractures were determined in 12 (4.3%) adults and eight (4.6%) children.

Open fractures were seen in 17 (6%) adults and three (1.7%) children. Of the 20 open fractures, 18 were in the lower extremities. When evaluated with the Gustilo classification, type 3c was detected in four patients in adults, two of which were treated with an external fixator, and free flap operation was performed by plastic surgery at the foot/ankle level. The other two patients with type 3C fracture at the level of the forearm and femur diaphysis underwent amputation due to non-revascularization despite vascular repair and external fixator.

Management

Operations were performed in the first 12 hours in 19 (13 M, 6 F) (6.8%) adult patients and 58 (33.3%) pediatric patients. The rate of emergency operations in pediatric patients was determined to be significantly high (p: 0.001). There were supracondylar humerus fractures in 46 of the 58 pediatric patients who underwent emergency surgery.

External fixators (EF), intramedullary nails (IMN), K-wire augmentation, cannulated screws, plate fixation (PF), and posterior instrumentation (PI) were used in the treatment of fractures. Whilst, PF was mostly used in adult patients (137, 48.8%), K-wire augmentation was used in pediatric patients (75, 43.1%). While fixation with plate was made in nine patients with pelvic fracture due to acetabulum fracture, sacral bridge plating-external fixator was applied to the remaining three patients with pelvic fractures. Posterior instrumentation was performed in patients with lumbar 1 and thoracic 12 vertebra compression fractures.

In the comparisons of the ASA scores of the patients in adults, the ASA scores of the female patients were found to be higher than those of males, since 22 (27.8%) of the female patients were aged >65 years.

Prognosis and outcome

The mean hospitalization period was 5.4 (5.1) days (range, 1-39 days) in adult patients and 3.1 (3.4) days (range, 1-36 days) in pediatric patients.

Out of the 455 patients, 41 (14.6%) adults and 13 (7.3%) children developed complications with more than one complication in some patients; in adult group, osteomyelitis in 10 (3.5%), non-union in 11 (3.9%), arthrosis in eight (2.8%), embolism/vascular problems in four (1.4%), malunion in two (0.7%), superficial infection in three (1%), ARDS in two (0.7%) and nerve injury in one (0.35%). In the pediatric group, complications were observed in 13 (7.3%) patients; six malunion (3.4%), six limitation of movement (3.4%) and one superficial infection (0.5%). The complication rate of the adult patients was found to be significantly high (p = 0.02).

When we compared with Turkish citizens of the same number, gender, and age range by making propensity score matching on the same dates, the complication rate was 54/455 in Syrian immigrants, while it was 32/455 in citizens, and it was observed that the complication was significantly higher in immigrants (p = 0.017).

Mortality developed in three patients. A 46-year-old male patient who was operated on for lumbar vertebrae fracture was applied with debridement due to early infection (P. aeruginosa) and died as a result of pulmonary embolism after debridement. Of the female patients, an 85-year-old with a femoral diaphyseal fracture and a 78-year-old with a femoral neck fracture were postop ex due to ARDS in the intensive care unit. No deaths were recorded in the pediatric patients.

When the postoperative outpatient controls were compared to evaluate the compliance with the treatments, it was seen that 65 (14.2%) of the Syrian immigrants did not come to the polyclinic control at all or once, while this rate was 29/455 (6.3%) for Turkish citizens. It was found statistically significant that Syrian immigrants did not come to the postop outpatient clinic compared to Turkish citizens (p = 0.012).

Comparing the cost analysis with the same number of patients, it was seen that it was 2,615,997 TL for Turkish citizens and 3,190,760 TL for Syrian refugees. The increase in the average cost was associated with worse prognosis, complications, intensive care treatments, and prolonged hospitalization periods.

## Discussion

Turkey is currently hosting the largest number of Syrian refugees - more than 2.7 million. A limited number of refugees are living in camps settled around the border, and others are spread throughout Turkey. This explosive and unexpected increase in the Syrian population in Turkey has had several negative impacts on health and social determinants [6]. The Turkish government has made several regulations for Syrian refugees, which allow them to benefit from emergency care units and primary, secondary, and tertiary healthcare centres in Turkey's 81 provinces. Effectiveness of healthcare services for refugees is limited by language barriers and mobility of the refugees [7]. Comorbidities were common, potentially having a negative influence on treatment processes. This can be a result of unfavorable living conditions and lack of medical care during migration.

In this report, we highlighted the challenges in management and follow-up by presenting the spectrum, management, and consequences of fracture surgeries in Syrian refugees. We also compared Turkish citizens with Syrian refugees and wanted to share the impact of living as a refugee on outcomes.

According to AFAD (Disaster and Emergency Management Authority) data, the gender distribution of Syrian refugees, in general, is almost equal (M: 50.80%-F: 49.20%). While the highest rate (52.20%) of patients was in the 19-54 years age group, followed by the 0-12 years group, and the lowest rate was in the ≥65 years age group [3,4]. In the present study, the rate of children (<18 years) was 174/455 (38.2%) and in the adults, 205/455 (45.1%) were between the ages of 18-54 years, and the rate of patients over 65 years was 40/455 (8.8%).

The demographic characteristics of victims of civil war are closely related to the social structure of the population. In previous studies, it has been reported that most refugees (70-100%) are male and young (16-34 years old) [8-10]. In accordance with the literature, in the present study, the mean age of the patients was 28.7 (22.0) years (range, 1-86 years), and the number of male patients was higher than the number of female patients (M: 72.5%, F: 27.5%). The high number of male refugees was thought to be because male refugees are working to support the family and are therefore more exposed to trauma.

When the mechanism of fractures is examined in the literature, the most common cause has been reported to be traffic accidents at rates of 63.6% [11] and 39.1% [12]. In the same study, it is stated that falls are the second most common determinant of traumatic orthopedic injuries at the rates of 21.8% [11] and 35.1% [12]. When the mechanism of the trauma of the patients in the current study was evaluated, the leading cause was falling (66.6%), followed by motor vehicle accidents, of which 19.1% were out of vehicle traffic accidents. In the present study, contrary to the literature, we think that the fall is in the first place and the motor vehicle accident is in the second place. Syrian refugees live in low socioeconomic living conditions and are employed illegally and without insurance.

In a study of 2817 patients, Taylor and Young [13] stated that lower limb fractures were the most common with 53.6%, with the average age of patients as 53.1 years and a male: female ratio of 51%:49%. In the current study, the male: female ratio was 72%:28% in adult patients and the leading injury was lower limb fractures in 287/455 (63.1%) cases, followed by upper limb fractures in 154/455 (33.8%) cases and the least frequent were pelvis/vertebral injuries seen in 14/455 (3.1%) cases. Although falling as the mechanism of trauma occurs in the first place among Syrian refugees, they had similar results to the literature in terms of gender and fracture site rates. For this reason, we think that the mechanism of trauma does not affect the fracture site. In addition, we believe that male gender dominance in the current study is also due to a much higher number of men in the refugee population and a higher proportion of male refugees in working life.

In the current study in accordance with the literature, the most common lower limb fracture was proximal femur fracture at the rate of 53/281 (18.86%) and this rate was higher in female patients. In epidemiological studies of adult fracture patients, Court-Brown and Caesar [14] reported proximal femur fracture at the rate of 11.6% (692/5953) and 74% of these patients were female. In the same study, pelvic fractures constituted 1.5% (91/5953), and vertebral fractures constituted 0.7% (40/5953) of all fractures. In the current study, 12/455 (2.63%) pelvic fractures and 2/455 (0.43%) vertebral fractures were observed. The lower incidence of vertebral fractures was attributed to the fact that spinal surgery was also performed by the Neurosurgery Department. In addition, we think that the high incidence of pelvic fractures is also due to the refugees working in heavy work (such as construction work).

Rennie et al. [15] reported that 10-25% of all childhood injuries were fractures. Another difference is that children mainly present with upper limb fractures and the rate of lower limb fractures is relatively low. Spinal and pelvic fractures are rare in both adults and children [1]. Supracondylar humerus fractures make up 17% of all childhood fractures and are generally seen at an average of 5-8 years old [16,17]. In the current study, the mean age of supracondylar humerus fracture cases was 5.22 years and was the most common site of surgery in children at the rate of 55/174 (31.6%). In accordance with the literature, upper limb fractures in children and lower limb fractures in adults were seen at significantly higher rates.

There are many patients who have difficulties in communicating with healthcare providers due to language barriers, who do not have sufficient medical records, and are socially and psychologically affected [18]. According to AFAD [3] data, 44.50% of Syrian refugees speak Turkish very badly or do not speak it at all. Therefore, communication and outpatient follow-up of patients can be difficult. While the rate of adult females and children coming to polyclinic follow-up appointments is high, this rate is low in adult males. One of the subjects of concern is that early intervention cannot be made to complications such as infection because of the problems in follow-up of these patients. Of the total 78 tibia fractures (nine open) in the current study, osteomyelitis developed in two (2.8%) patients with a closed fracture and in three (33.3%) with an open fracture. Doshi et al. [19] found a low incidence of infections following surgical management of tibia fractures in a cohort of 787 participants in India in their prospective cohort study. The incidence of infection for closed and open fractures was 1.6% and 8.0%, respectively. The higher rates seen in the current study can be attributed to poor nutrition and not attending regular polyclinic follow-up.

Various factors affect mortality in hip surgeries, mostly hip fractures in geriatric patients. In particular, the ASA score affects morbidity and mortality in hip surgery [20]. Of 23 hip fracture patients in the current study, two were exitus: both had an ASA score of 3, and both were treated in the intensive care unit for four days.

In the literature, in studies in which fracture patients operated in orthopedic surgery were screened, Platz and Hyman [21] reported 13% of the complication rates, and Meeuwis et al. [22] also stated the complication rate as 19.8%. In the present study, the complication rate was found to be 14.6% in adult patients and 7.3% in pediatric patients. The high complication rate in adult patients compared to pediatric patients is thought to be due to the fact that these patients do not attend outpatient clinic follow-ups as often as pediatric patients.

Limitations

As the study data were retrospectively analyzed, other factors affecting the outcome, such as co-morbidities, requested medical consultations, use of an interpreter, and departure status between admittance and discharge could not be evaluated.

## Conclusions

Poor living conditions and malnutrition increase the complication rate, length of hospitalization, and cost of Syrian refugees. Lack of communication reduces the rate of patient follow-up, consequently, the results of orthopedic trauma are negatively affected.

## References

[1] Devi S (2016). Syria’s health crisis: 5 years on. Lancet.

[2] (2021). United Nations High Commissioner for Refugees. Forced displacement in 2017. https://www.unhcr.org/globaltrends2017/.

[3] (2021). Syria reports. Türkiye’deki Suriyelilerin Demograﬁk Görünümü, Yașam Koșulları ve Gelecek Beklentilerine Yönelik Saha Araștırması.

[4] (2016). Syrian refugees in Turkey. Syrian Refugees in Turkey.

[5] Gustilo RB, Mendoza RM, Williams DN (1984). Problems in the management of type III (severe) open fractures: a new classification of type III open fractures. J Trauma Acute Care Surg.

[6] Doganay M, Demiraslan H (2016). Refugees of the Syrian civil war: impact on reemerging infections, health services, and biosecurity in Turkey. Health Secur.

[7] Assi R, Ozger-Ilhan S, Ilhan MN (2019). Health needs and access to health care: the case of Syrian refugees in Turkey. Public Health.

[8] Çelikel A, Karaarslan B, Demirkıran DS, Zeren C, Arslan MM (2014). A series of civilian fatalities during the war in Syria. Ulus Travma Acil Cerrahi Derg.

[9] Weerasuriya CK, Tan SO, Alexakis LC (2016). Evaluation of a surgical service in the chronic phase of a refugee camp: an example from the Thai-Myanmar border. Confl Health.

[10] Buz S (2008). The social profile of asylum seekers in Turkey. Turk J Police Stud.

[11] Huda N, Gupta P, Pant A, Iqbal A, Julfiqar M, Khan MZ, Agrawal NK (2014). Pattern of orthopaedic injuries among patients attending the emergency department in a tertiary care hospital - An analytical study. Acta Medica Int.

[12] Ahmed E, Chaka T (2005). Orthopedic and major limb trauma at the Tikur Anbessa University Hospital, Addis Ababa -Ethiopia. East Cent Afr J Surg.

[13] Taylor A, Young A (2015). Epidemiology of orthopaedic trauma admissions over one year in a district general hospital in England. Open Orthop J.

[14] Court-Brown CM, Caesar B (2006). Epidemiology of adult fractures: a review. Injury.

[15] Rennie L, Court-Brown CM, Mok JY, Beattie TF (2007). The epidemiology of fractures in children. Injury.

[16] Houshian S, Mehdi B, Larsen MS (2001). The epidemiology of elbow fracture in children: analysis of 355 fractures, with special reference to supracondylar humerus fractures. J Orthop Sci.

[17] Barr LV (2014). Paediatric supracondylar humeral fractures: epidemiology, mechanisms and incidence during school holidays. J Child Orthop.

[18] Ozdogan HK, Karateke F, Ozdogan M, Satar S (2014). Syrian refugees in Turkey: effects on intensive care. Lancet.

[19] Doshi P, Gopalan H, Sprague S, Pradhan C, Kulkarni S, Bhandari M (2017). Incidence of infection following internal fixation of open and closed tibia fractures in India (INFINITI): a multi-centre observational cohort study. BMC Musculoskelet Disord.

[20] Akcan A, Şanal Baş S, Güleç MS (2020). Effect of regional or general anesthesia methods on mortality according to age groups in geriatric hip surgery patients. Agri.

[21] Platz J, Hyman N (2012). Tracking intraoperative complications. J Am Coll Surgeons.

[22] Meeuwis MA, de Jongh MA, Roukema JA, van der Heijden FH, Verhofstad MH (2016). Technical errors and complications in orthopaedic trauma surgery. Arch Orthop Trauma Surg.

